# Draft genome sequences of *Flagellimonas* sp. MMG031 and *Marinobacter* sp. MMG032 isolated from the dinoflagellate *Symbiodinium pilosum*

**DOI:** 10.1128/mra.00913-24

**Published:** 2024-12-10

**Authors:** Morgan V. Farrell, Aya M. Aljaber, Madison Amoruso, Wyman F. Chan, Jiellen R. Dael, Mathieu L. De Tomas, Emily G. Delavega, Jacob M. Eslava, Benjamin J. Holdbrook-Smith, Precilla Lee, Van Mai, Laith R. Michael, Stephanie V. Moreno, Jose F. Quevedo, Aaron G. Roberts, Jorge Villanueva, Carl Westin, Daniela M. Zazueta, Nicholas J. Shikuma

**Affiliations:** 1 Department of Biology and Viral Information Institute, San Diego State University, San Diego, California, USA; DOE Joint Genome Institute, Berkeley, California, USA

**Keywords:** symbiosis, marine microbiology, coral

## Abstract

Here, we report the draft genome sequences of *Flagellimonas* sp. MMG031 and *Marinobacter* sp. MMG032, isolated from coral-associated dinoflagellate *Symbiodinium pilosum*, assembled and analyzed by undergraduate students participating in a Marine Microbial Genomics (MMG) course. A genomic comparison suggests MMG031 and MMG032 are novel species and a resource for restoration and biotechnology.

## ANNOUNCEMENT

To engage undergraduates in discovery-based research, novel marine bacteria were isolated and cultured, their genomes sequenced, assembled, annotated, and analyzed by students in a Marine Microbial Genomics (MMG) course at San Diego State University. Strains MMG031 and MMG032 were isolated from the coral microalgal symbiont *Symbiodinium pilosum* (1), purchased from Bigelow National Center for Marine Algae (East Boothbay, ME, USA). Bacterial cultures were plated and isolated (three times) on Marine Broth (MB) 2216 (BD Difco, Franklin Lakes, NJ, USA) agar plates for 72 hours at 25°C. A single bacterial colony was inoculated into the MB liquid and incubated for 24 hours at 25°C before storage and DNA isolation.

Genomic DNA was extracted using a Quick-DNA Fungal/Bacteria Miniprep Kit (Zymo Research, Irvine, CA, USA). DNA was submitted to the Plasmidsaurus Sequencing Center (Eugene, OR, USA) for library preparation (Oxford Nanopore Rapid Sequencing Kit V14) and whole genome sequencing (Oxford Nanopore Sequencing, R10.4.1 flow cell), producing 172,285 total reads and a median read length of 10 kb for MMG031, and 94,537 total reads and a median read length of 5.7 kb for MMG032. Reads were trimmed using Fitlong v0.2.1 (2) assembled with Flye v2.9.3 (3) and polished with Racon v1.4.20 (4) to determine if any contigs were circular by identifying overlapping regions. Genome annotation was performed with Prokka v1.14.5 (5) and the NCBI Prokaryotic Genome Annotation Pipeline (PGAP) v5.1 (6). MMG031 has a 3.8-Mb genome, a GC content of 43.5% with one contig at 311× coverage, a contig *N*_50_ value of 3.8 Mb, and 3,433 predicted coding sequences. MMG032 has a 4.3-Mb genome, a GC content of 57.5% with two contigs at 131× coverage, a contig *N*_50_ value of 4.2 Mb, and 3,992 predicted coding sequences. Default parameters were used except when noted.

**Fig 1:**
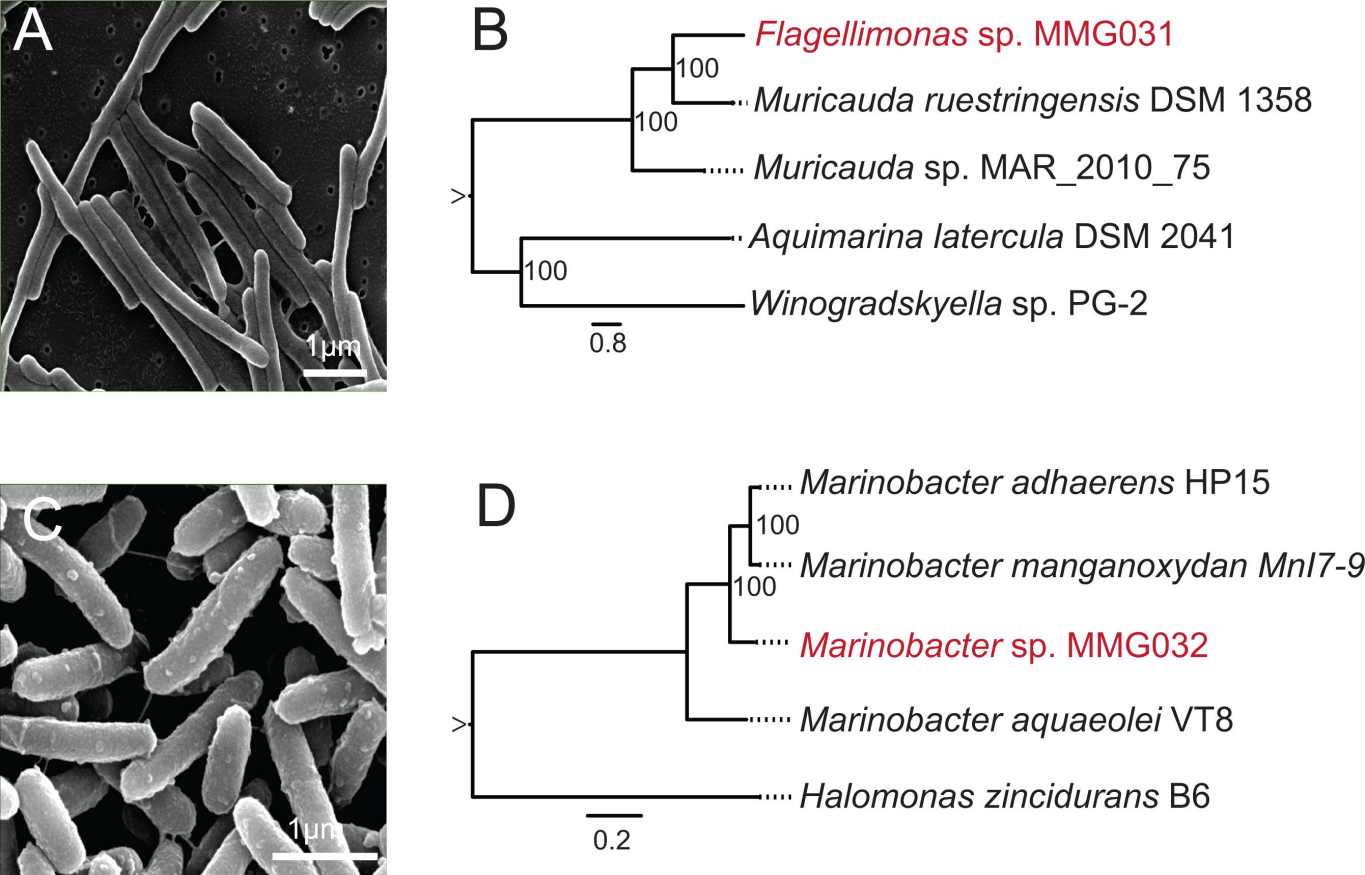
**(A**) Scanning electron microscopy (SEM) (Model Quanta 450 FEG) image of *Flagellimonas* sp. MMG031. Bacterial samples were fixed with 2.5% glutaraldehyde (Sigma-Aldrich, G5882) overnight at 4°C, then dehydrated onto a 0.2-µm membrane (Sigma-Aldrich, GTBP01300). (**B**) A maximum likelihood phylogeny of *Flagellimonas* sp. MMG031 was constructed using the codon tree method through PATRIC using 100 single-copy genes and proteins identified by PGFams (13–19). Newly isolated strain *Flagellimonas* sp. MMG031 in red. GenBank accession numbers for the phylogenetic tree are as follows: *Muricauda ruestringensis* DSM 13258 (CP002999)*, Muricauda* sp. MAR_2010_75 (JQNJ00000000), *Aquimarina latercula* DSM 2041 (AUMK00000000), *Winogradskyella* sp. PG-2 (AP014583.1). (**C**) SEM image of *Marinobacter* sp. MMG032. (**D**) A maximum likelihood phylogeny of *Marinobacter* sp. MMG032 was constructed using the codon tree method through PATRIC using 100 single-copy genes and proteins identified by PGFams (6, 13–18, 20, 21). Newly isolated strain *Marinobacter* sp. MMG032 in red. GenBank accession numbers for the phylogenetic tree are as follows: *Marinobacter adhaerens* HP15 (CP001978, CP001979, CP001980); *Marinobacter manganoxydans* MnI7-9 (AGTR00000000); *Marinobacter aquaeolei* VT8 (CP000514, CP000516, CP000515); *Halomonas zincidurans* B6 (JNCK00000000); *Halomonas* sp. S2151 (JXYB00000000).

A phylogenetic analysis revealed that strain MMG031 belongs to the genus *Flagellimonas* (formerly *Muricauda* [7], Fig. 1)*,* family *Flavobacteriaceae*, and class Flavobacteriia (8, 9). Strain MMG032 belongs to the genus *Marinobacter*, family *Marinobacteraceae*, and class Gammaproteobacteria (10). Comparing strain MMG031 with *Muricauda ruestringensis* DSM 13258 yields an average nucleotide identity (ANI, CJ Bioscience) value of 79.74% (5, 11). Comparing strain MMG032 with *Marinobacter manganoxydans* Mnl7-9, the average ANI was 86.65%. The ANI values of strains MMG031 and MMG032 are significantly below the 95% threshold that delineates species (12), suggesting that they are novel isolates. We designate the isolates *Flagellimonas* sp. strain MMG031, and *Marinobacter* sp. strain MMG032.

Other species of *Flagellimonas* (or *Muricauda*) and *Marinobacter* are beneficial microbes associated with thermotolerant microalga (22–24) that can improve stress tolerance (25). The analysis conducted using AntiMASH (26) revealed *Marinobacter* sp. MMG032 has gene clusters for ectoine, a putative thermoprotectant (27). *Flagellimonas* sp. MMG031 genome analysis revealed gene clusters for lycopene beta-cyclase, an enzyme involved in the biosynthesis of vitamin A, a potent antioxidant that protects *Symbiodinium* from heat and light stress (20, 21). Further investigation of the association between *Symbiodinium* algae and *Flagellimonas* sp. MMG031 or *Marinobacter* sp. MMG032 could lead to insights into coral symbiosis.

## Data Availability

The genome sequencing and assembly project for strains MMG031 and MMG032 have been deposited in DDBJ/EMBL/GenBank under BioProject number PRJNA716944, raw sequencing SRA accession numbers SRX24549989 and SRX24549990, respectively, and assembly accession of GCF_040112705.1 and GCF_040112695.1, respectively.
